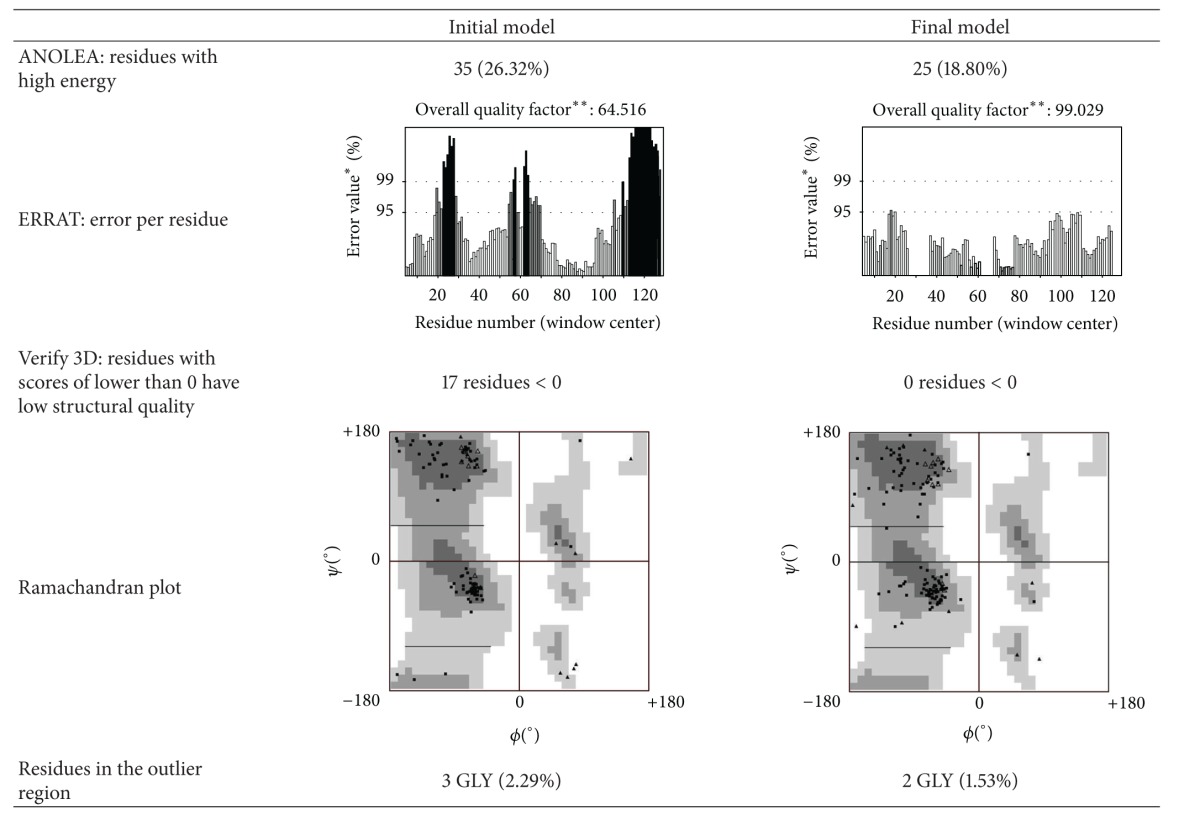# A Novel Phospholipase A_**2**_ (D49) from the Venom of the *Crotalus oreganus abyssus* (North American Grand Canyon Rattlesnake)

**DOI:** 10.1155/2014/654170

**Published:** 2014-02-24

**Authors:** W. Martins, P. A. Baldasso, K. M. Honório, V. G. Maltarollo, R. I. M. A. Ribeiro, B. M. A. Carvalho, A. M. Soares, L. A. Calderon, R. G. Stábeli, M. A. O. Caballol, G. Acosta, E. Oliveira, S. Marangoni, F. Albericio, S. L. Da Silva

**Affiliations:** ^1^Department of Chemistry, Biochemistry and Bioprocess Engineering, Federal University of São João Del Rei, Campus Alto Paraopeba, 34420-000 Ouro Branco, MG, Brazil; ^2^Department of Biochemistry of Biology Institute, State University of Campinas, 13083-970 Campinas, SP, Brazil; ^3^School of Arts, Sciences and Humanities, University of São Paulo, 03828-000 São Paulo, Brazil; ^4^Center of Natural Sciences and Humanities, Federal University of ABC, 09210-170 Santo Andre, SP, Brazil; ^5^Center of Biomolecules Study Applied to Health, Fiocruz Rondônia, Oswaldo Cruz Foundation, Medicine Department, Federal University of Rondônia, 76812-245 Porto Velho, Brazil; ^6^Proteomics Platform, Barcelona Science Park, University of Barcelona, Baldiri Reixac, 08208 Barcelona, Spain; ^7^Institute for Research in Biomedicine, Barcelona Science Park, University of Barcelona, 08280 Barcelona, Spain; ^8^CIBER-BBN, Barcelona Science Park, University of Barcelona, 08280 Barcelona, Spain; ^9^Department of Organic Chemistry, University of Barcelona, 08028 Barcelona, Spain; ^10^School of Chemistry and Physics, University of KwaZulu-Natal, Durban 4001, South Africa

## Abstract

Currently, *Crotalus viridis* was divided into two species: *Crotalus viridis* and *Crotalus oreganus*. The current classification divides “the old” *Crotalus viridis* into two new and independent species: *Crotalus viridis* (subspecies: *viridis and nuntius*) and *Crotalus oreganus* (subspecies: *abyssus, lutosus, concolor, oreganus, helleri, cerberus, and caliginis*). The analysis of a product from cDNA (E6d), derived from the gland of a specie *Crotalus viridis viridis*, was found to produce an acid phospholipase A_2_. In this study we isolated and characterized a PLA_2_ (D49) from *Crotalus oreganus abyssus* venom. Our studies show that the PLA_2_ produced from the cDNA of *Crotalus viridis viridis* (named E6d) is exactly the same PLA_2_ primary sequence of amino acids isolated from the venom of *Crotalus oreganus abyssus*. Thus, the PLA_2_ from E6d cDNA is actually the same PLA_2_ presented in the venom of *Crotalus oreganus abyssus* and does not correspond to the venom from *Crotalus viridis viridis*. These facts highlight the importance of performing more studies on subspecies of *Crotalus oreganus* and *Crotalus viridis*, since the old classification may have led to mixed results or mistaken data.

## 1. Introduction

**Figure 1:**
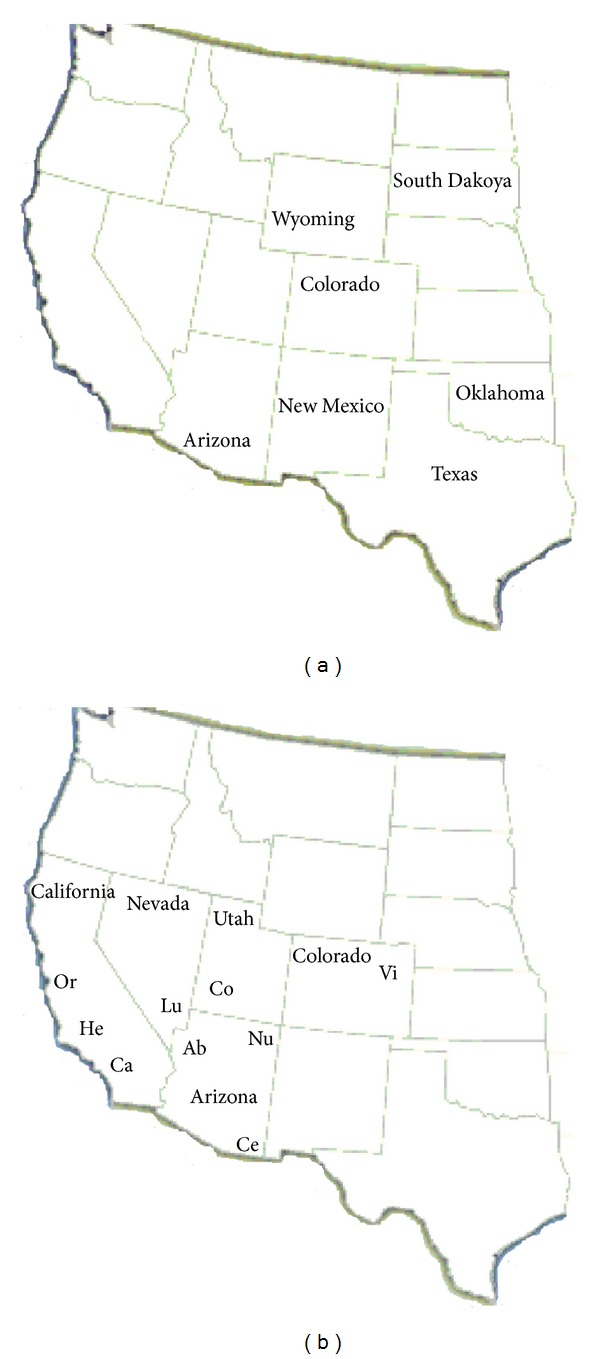
States inhabited by subspecies of *C. viridis* and *C. oreganus*. (a) States from which snakes used by Tsai et al. [37] were collected. (b) States and approximate locations from which snakes were collected Mackssey [2].

*Crotalus viridis* defines a large group of snakes, also named as Western Rattlesnakes, which inhabit the eastern region of the Rocky Mountains of the United States that stretch from southern Canada to northern Mexico (Figure 1) [1, 2]. Phylogenetic analyses on mitochondrial DNA sequences of snakes classified as *Crotalus viridis* show significant taxonomic variations between individuals from different areas of USA and indicate that this species has several subspecies [3]. To understand the variations in these *subspecies*, morphological analyses were carried out based on distance analysis of whole venom profiles and based on maximum parsimony (MP) analysis of cyt *b* and ND4 [1, 4, 5].

Initially, based only on morphology, Klauber classified these snakes into nine subspecies of *Crotalus viridis: C. v. viridis, C. v. nuntius, C. v. abyssus, C. v. lutosus, C. v. concolor, C. v. oreganus, C. v. helleri, C. v. Cerberus, and C. v. caliginis* [1]. This classification was in place until the early 2000s, when reports by Pook et al. and Asthon and de Queiroz, based on the analysis of the molecular characteristics of DNA of the nine subspecies of *C. viridis*, showed that the subspecies could be grouped into two distinct and new groups: *Crotalus viridis* and *Crotalus oreganus* [3–5]. The new classification, grouped the *C. viridis* into two new subspecies: *C. v. viridis* and *C. v. nuntius* and *Crotalus oreganus* into five new subspecies: *C. o. abyssus, C. o. lutosus, C. o. concolor, C. o. oreganus, C. o. helleri, C. o. Cerberus, and C. o. caliginis*. This is the current and official classification used for snakes previously classified as *C. viridis* (and its subspecies).

Secreted phospholipases A_2_ (PLA_2_s) are a family of relatively stable enzymes, with low molecular mass (13–15 kDa) and 6 (or 7) conserved disulfide bonds. PLA_2_s employ calcium ions and the amino acid residues, Asp47, and His48, to catalyze the hydrolysis of the *Sn-2* of glycerophospholipid esters bonds of membranes. This hydrolysis reaction releases glycerol and proinflammatory eicosanoids [6–10].

PLA_2_ are present in snake venom and the biological fluids, cells, and tissues of these species and are widely studied due to their pharmacological diversity. These enzymes can act as regulators of the membrane phospholipid membrane homeostasis [8–10] and also present physiopathological processes that can be neurotoxic (pre- or postsynaptic), cardiotoxic [11–14], hypotensive [15–17], anticoagulant and platelet aggregating [18, 19], genotoxic [20, 21], myotoxic [22, 23], antitumoral, and bacterial [24, 25]. Due to the toxic pharmacological effects produced by PLA_2_, several studies have researched or developed natural or synthetic compounds to aid in the treatment of the snake bites to inhibit the toxic effects of PLA_2_ [26–33]. In addition, the amino acid sequences of hundreds of PLA_2_s from snake venom have been determined [34–36].

Tsai et al. studied PLA_2_ from glands obtained from different samples of *C. viridis viridis,* arising from several regions of the United States (Figure 1(a)) [37]. They purified and sequenced five acidic PLA_2_s sharing 78% or greater sequence identity. Interestingly, Tsai et al. observed that the product of the cDNA sequence named cvvE6d modified a PLA_2_ with a molecular mass of 13782 ± 1 Da. This specific molecule of PLA_2_ was found only in a unique snake from Southeastern Arizona. The authors correctly inferred and suggested that these individuals from Southeastern Arizona could actually represent a distinct population of *Crotalus viridis viridis *[37].

Recently, while studying the differences in total venoms from *C. viridis* and *C. oreganus* subspecies, Mackessy verified that all venoms display great variation, both in protein composition as well as in the activities of several enzymes, including the PLA_2_ enzyme family [2]. The venom used by Mackssey was obtained from *C. viridis* and *C. oreganus* subspecies from the locations shown in Figure 1(b) [2].

According to Mackssey, as the Western Rattlesnake occurs across a broad geographical area, it represents an ideal species group to investigate variations in venom composition, and to understand how these differences evolve and how composition affects the biological role(s) of venom [2]. In this study, to further the understanding of the biological diversity of the subspecies of *C. oreganus,* we biochemically isolated and characterized a PLA_2_ (D49) from *C. oreganus abyssus* venom. Moreover, we sequenced the primary structure of PLA_2_, performed pharmacological and biochemical characterization assays, and used molecular modeling to analyze the structure obtained.

## 2. Material and Methods

### 2.1. Material

All reagents were purchased from Aldrich or Sigma Co (USA). *Crotalus oreganus abyssus* (Coa), *Crotalus viridis viridis* (Cvv), and *Crotalus viridis nuntius* (Cvn) venoms were obtained from The National Natural Toxins Research Center (NNTRC) of Texas A&M University-Kingsville (Kingsville, Texas, USA). The substrate, 1-hexadecanoyl-2-(1-pyrenedecanoyl)-sn-glycero-3-phosphoglycerol (HPGP), was supplied by Molecular Probes (USA). The substrate 4-nitro-3-octanoyloxy benzoic acid (NOBA) was synthesized following the methodology described by Cho et al. [38].

### 2.2. Isolation of the Phospholipase A_2_ from *Crotalus oreganus abyssus *(CoaPLA_2_)

Venom from *C. o. abyssus* (200 mg) was fractioned by chromatography on a G75-Sephadex column, previously balanced with 0.05 M ammonium bicarbonate buffer (AMBIC—pH 8.0). Elution was performed using 1.0 M ammonium bicarbonate (AMBIC—pH 8.0) at a flow rate of 0.5 mL/min. Fraction II, presenting phospholipase activity, was collected and ultrafiltered using the MidJet apparatus (Ge Healthcare, USA) equipped with the UFP-10-C-MM01A cartridge (superficial area of 26 cm^2^, cut off: 10,000 Da—Ge Healthcare, USA). The filtrate was lyophilized and stored frozen at −20°C.

Lyophilized fraction II (25 mg), containing PLA_2_ activity, was dissolved in 250 *μ*L of 5% (v/v) acetonitrile in 0.1% (v/v) trifluoroacetic acid (TFA), homogenized and centrifuged at 480 ×g for 5 min, and then subjected to a reverse phase HPLC (model 2010, Shimadzu, Japan) using an analytical C18 column (Supelco, 250 mm × 4.6 mm). The analytical C18 column was equilibrated in solvent A (5% acetonitrile, 0.1% TFA) and elution proceeded with a concentration gradient from 0 to 100% of solvent B (60% acetonitrile, 0.1% TFA), at a flow rate of 1 mL/min, for 60 min.

**Figure 2:**
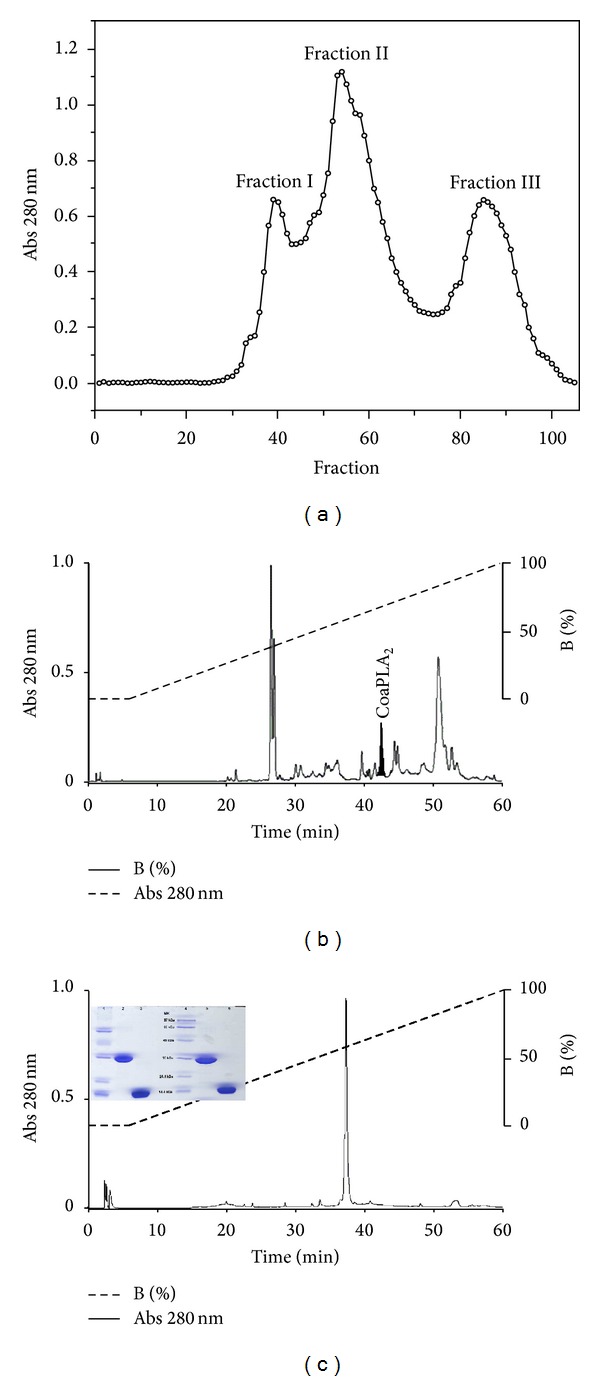
Isolation of CoaPLA_2_ from *Crotalus oreganus abyssus* venom. (a) Profile obtained by gel chromatography on a G75-Sephadex column. Fraction II, presenting phospholipase activity. (b) Lyophilized fraction II was homogenized and centrifuged and subjected to reverse phase HPLC using an analytical C18 column. (c) Assessment of purity of RP-HPLC under the same conditions used in (b). Electrophoretic analysis shows the homogeneity of the CoaPLA_2_ isolated from the venom of *Crotalus Oreganus abyssus*: Lines 1 and 4: standard molecular weight; Lines 2 and 5: PAGE under nonreduced conditions; Lines 3 and 6: PAGE under reduced conditions (SDS-PAGE).

To help remove any other impurities that might be present, the fraction with PLA_2_ activity was again subjected to ultrafiltration using the MidJet apparatus (Ge Healthcare, USA), equipped with the UFP-10-C-MM01A cartridge (superficial area of 26 cm^2^, cut off: 10,000 Da—Ge Healthcare, USA). A PLA_2_ named CoaPLA_2_ was isolated, and the filtrate was lyophilized and rechromatographed to evaluate its purity, under the same conditions as described above (Figure 2). The fractions were monitored by spectrophotometry at 280 nm. The purity level of the CoaPLA_2_ was also evaluated using native polyacrylamide gel (PAGE) and sodium dodecyl sulfate-polyacrylamide gel electrophoresis (SDS-PAGE) [18, 21–23].

### 2.3. Biochemical Characterization of CoaPLA_2_

#### 2.3.1. SDS-PAGE and PAGE Electrophoresis

Electrophoresis analysis was performed to evaluate the purity and estimate molecular mass of CoaPLA_2_, under reducing and nonreducing conditions. The standard molecular weight proteins were purchased from BioRad Co. (Phosphorylase b—97,400; Serum albumin—66,200; Ovalbumin—45,000; Carbonic anhydrase—30,000; Trypsin inhibitor—20,100; Lysozyme—14,400 MW). CoaPLA_2_ pI was determined by isoelectric focusing, according to a previously described method [24–26].

#### 2.3.2. Phospholipase A_2_ Activity

Enzymatic activity was measured by two methods using two different substrates; a nonmicellar (4-nitro-3-octanoyloxy benzoic acid—NOBA) and a micellar substrate (1-hexadecanoyl-2-(1-pyrenedecanoyl)-sn-glycero-3-phosphoglycerol—HPGP).

*(1) Phospholipase A*_*2*_ *Activity Measured Using a Nonmicellar Substrate (4-nitro-3-octanoyloxy benzoic acid*—*NOBA)*. The phospholipase A_2_ activity of CoaPLA_2_ (both in the isolated protein and in total venoms) was measured using the assay described by Holzer and Mackessy [43], but modified for 96-well plates [17, 31–34]. The standard assay mixture contained 200 *μ*L of buffer (10 mM Tris-HCl, 10 mM CaCl_2_ and 100 mM NaCl, pH 8.0), 20 *μ*L of substrate (3 mM 4-nitro-3-octanoyloxy benzoic acid), 20 *μ*L of water, and 20 *μ*L of PLA_2_ (10 mg/mL) in a final volume of 260 *μ*L. After adding PLA_2_ (or total venom) (20 *μ*g), the mixture was incubated for up to 40 min at 37°C, with the reading absorbance at intervals of 10 min until 60 min. Enzyme activity, expressed as the initial velocity of the reaction (*V*_0_), was calculated based on the of absorbance at 20 min. After this time, the velocity did not change (maximum velocity was achieved). Enzyme activity was expressed as mean ± SD of three independent experiments and each experiment was carried out in triplicate. 

*(2) Phospholipase A*_*2*_ *Activity Measured Using a Micellar Substrate (1-hexadecanoyl-2-(1-pyrenedecanoyl)-sn-glycero-3-phosphoglycerol*—*HPGP)*. The measurements of enzymatic activity using the substrate 1-hexadecanoyl-2-(1-pyrenedecanoyl)-sn-glycero-3-phosphoglycerol (HPGP) were carried out using a microtiter plate assay [10, 20, 44]. One hundred *μ*L of solution A in assay buffer (27 *μ*M bovine serum albumin, 50 mM KCl, 1 mM CaCl_2_, 50 mM Tris-HCl pH 8.0) were added to a 96-well microtiter plate. Solution B presented the same composition as Solution A but with PLA_2_ (0.5 *μ*g/mL) or total venom (1.0 *μ*g/mL) and was delivered in 100 *μ*L portions to four wells, except for the first one. As a control, instead of Solution B, an additional 100 *μ*L of Solution A was added to the first of the four wells in the assay. Solution B was prepared immediately prior to each set of assays to avoid loss of enzymatic activity. After the addition of Solution B, the assay was rapidly initiated by the addition of 100 *μ*L of Solution C (420 mM 1-hexadecanoyl-2-(1-pyrenedecanoyl)-sn-glycero-3-phosphoglycerol vesicles in assay buffer) with a repeating pipette to all four wells. The fluorescence (excitation = 342 nm, emission = 395 nm) was read with a microtiter plate spectrophotometer (Fluorocount, Packard Instruments). Enzyme activity, expressed as the initial velocity of the reaction (*V*_0_) was calculated based on the absorbance at 20 min. After this time, the velocity did not change (the maximum velocity was achieved). Enzyme activity was expressed as mean ± SD of three independent experiments and each experiment was carried out in triplicate.

#### 2.3.3. Optimal pH and Temperature Determination of the Enzymatic Activity

Optimal pH and optimal temperature of the PLA_2_ activity (using methodology described in Section 2.3.2(1)) of the CoaPLA_2_ were determined by incubating the enzyme in four buffers of different pH values (4–10) and at different temperatures (25, 30, 35, 40, and 45°C), respectively, as described above (Section 2.3.2). The effect of substrate concentration (10, 5, 2.5, 1.25, 0.625, and 0.312 *μ*M) on enzyme activity was determined by measuring the increase of absorbance after 20 min and absorbance values at 425 nm were measured with a VersaMax 190 multiwell plate reader (Molecular Devices, S., CA). Enzyme activity was expressed as mean ± SD of three independent experiments and each experiment was carried out in triplicate.

#### 2.3.4. Determination of Influence of Ca^2+^ (and Other Ions) on PLA_2_ Activity

Three experiments were carried out to determine the influence of calcium ions on CoaPLA_2_ activity (using methodology described in Section 2.3.2(1)). The activity was described above (Section 2.3.2). Initially, Ca^2+^ concentrations of 0, 1, and 10 mM were used. After this procedure, the other three experiments were carried out: (1) without Ca^2+^, but in the presence of 10 mM of Mg^2+^, Cd^2+^, and Mn^2+^; (2) 1 mM of Ca^2+^ in the presence of 10 mM of Mg^2+^, Cd^2+^, and Mn^2+^, and (3) 10 mM of Ca^2+^ in the presence of 10 mM of Mg^2+^, Cd^2+^, and Mn^2+^. The influences of the ions on the enzyme activity were measured by determining absorbances at 425 nm with a VersaMax 190 multiwell plate reader (Molecular Devices, S., CA). Enzyme activity was expressed as mean ± SD of three independent experiments and each experiment was carried out in triplicate.

### 2.4. Biological Activity

#### 2.4.1. Animals

Groups of 6 Swiss male mice (6–8 weeks old) were matched for body weight (18–22 g). The animals were housed for at least one week before the experiment in laminar-flow cages maintained at a temperature of 22 ± 2°C and a relative humidity of 50–60%, under a 12 : 12 h light-dark cycle. The animal experiments were carried out with the approval of the Institutional Ethics Committee, in accordance with protocols following the recommendations of the Canadian Council on Animal Care. The mice used in this study were kept under specific pathogen-free conditions.

#### 2.4.2.  50% Lethal Dose

**Figure 3:**
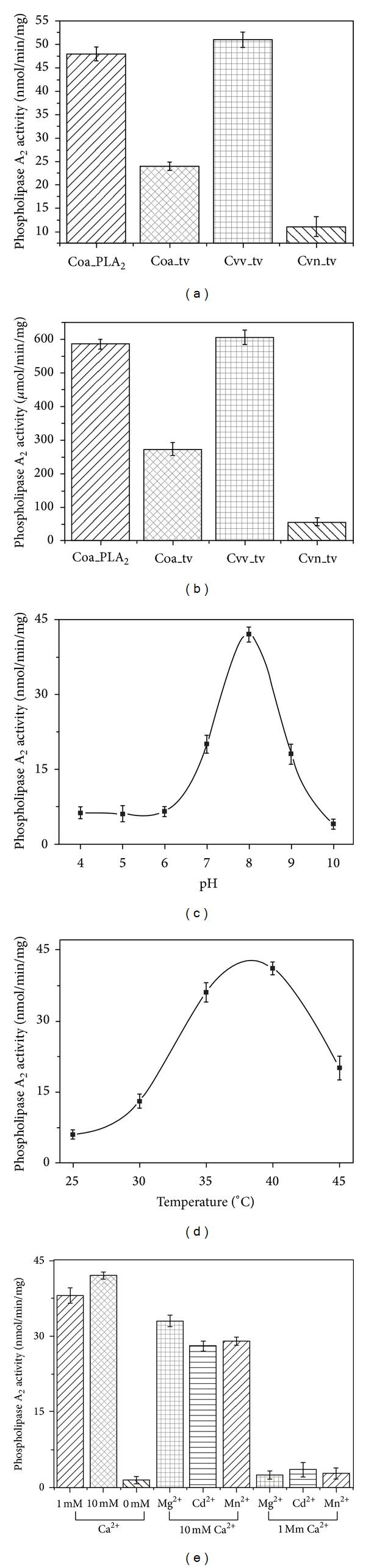
Phospholipase A_2_ activities of CoaPLA_2_ from *Crotalus Oreganus abyssus*. (a) Phospholipase A_2_ activities of CoaPLA_2_ and total venom of *Crotalus Oreganus abyssus*, *Crotalus viridis viridis,* and *Crotalus viridis nuntius* using a nonmicellar substrate (4-nitro-3-octanoyloxy benzoic acid); (b) Phospholipase A_2_ activities of CoaPLA_2_ and total venom of *Crotalus Oreganus abyssus*, *Crotalus viridis viridis,* and *Crotalus viridis nuntius* using a micellar substrate (1-hexadecanoyl-2-(1-pyrenedecanoyl)-sn-glycero-3-phosphoglycerol); (c) influence of pH variations on the enzymatic activity of CoaPLA_2_; (d) influence of temperature variations on the enzymatic activity of CoaPLA_2_; (e) analysis of the influence of calcium ions and other divalent cations on the phospholipase A_2_ activity of CoaPLA_2_. Results are expressed as mean ± SD of three independent experiments performed in triplicate (*n* = 3).

To evaluate the 50% lethal dose (dose that causes death in 50% of animals) of CoaPLA_2_ and the total venoms from *C. o. abyssus*, *C. v. viridis,* and *C. v. nuntius*, groups of six Swiss male mice (18−22 g) received an intravenous injection of 100 *μ*g of enzyme or total venom, dissolved in 100 *μ*L of PBS. As a control, six mice were similarly injected with 100 *μ*L of PBS alone. Animals were observed for up to 24 h after injection to record deaths. Lethal dose (LD) was expressed as mean ± SD of three independent experiments, performed in triplicate (*n* = 6) (Figure 3) [17, 18, 28, 29, 31, 33, 34].

#### 2.4.3. Edema-Inducing Activity

**Figure 4:**
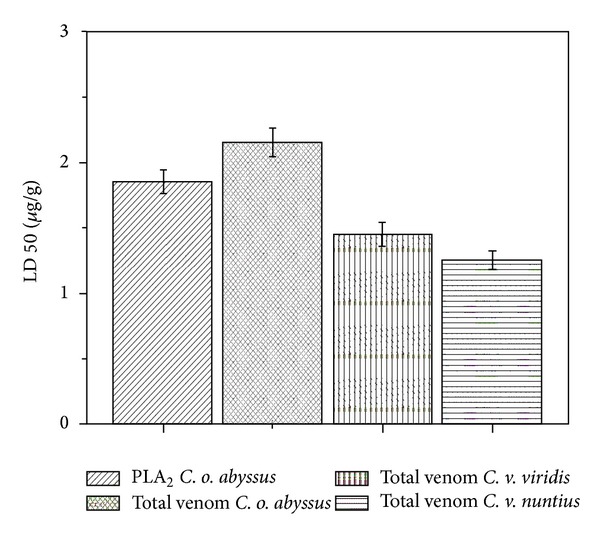
Lethal dose (dose that causes death in 50% of animals) of CoaPLA_2_ and total venoms of *Crotalus oreganus abyssus*, *Crotalus viridis viridis,* and *Crotalus viridis nuntius*. Lethal dose (LD) is expressed as mean ± SD of three independent experiments performed in triplicate (*n* = 6).

Groups of six Swiss male mice (18–22 g) were injected in the subplantar region with various amounts of total venom or CoaPLA_2_ (in a volume of 50 *μ*L) prepared in PBS, pH 7.2. Subsequently, paw volume was measured at different time intervals (30, 60, 120, and 180 min), subtracting the initial paw volume (time 0 h). Paw edema was measured with a low-pressure pachymeter (Mitutoyo, Japan). Edema-inducing activity was expressed as mean ± SD of three independent experiments and each experiment was carried out in triplicate (*n* = 6) (Figure 4) [17, 18, 28, 29, 31, 33, 34].

#### 2.4.4. Myotoxic Activity

**Figure 5:**
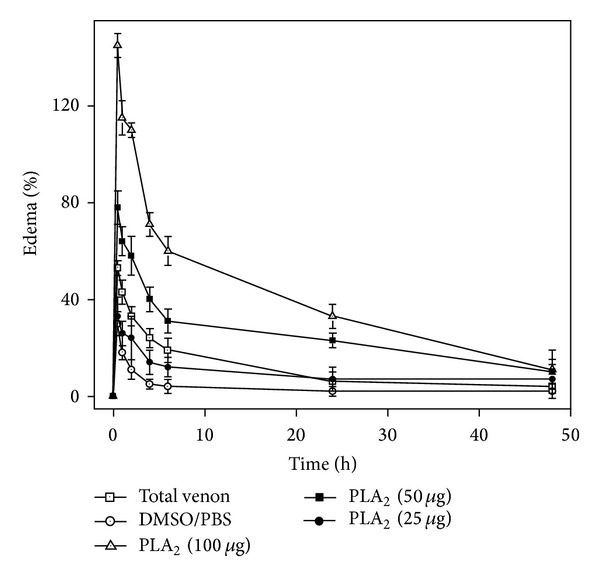
Mice paw edema induced by different doses of CoaPLA_2_. Positive control was the total venom of *Crotalus Oreganus Abyssus* and negative control was DMSO/PBS solution. Edema was expressed as the percentage increase in the paw volume of the treated group, compared to that of the control group at each time interval. Edema-inducing percentage is expressed as the mean ± SD of three independent experiments performed in triplicate (*n* = 6).

**Figure 6:**
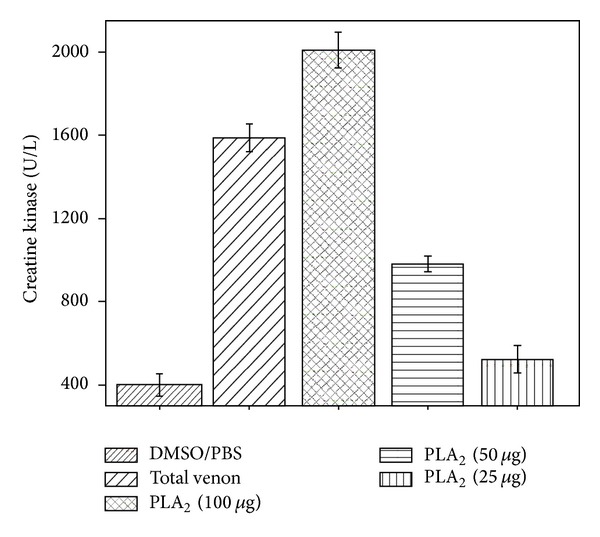
Myotoxic activity induced by different doses of CoaPLA_2_ from *Crotalus Oreganus Abyssus*. Negative control was PBS solution containing 1% of DMSO. Myotoxic activity was expressed as mean ± SD of three independent experiments performed in triplicate (*n* = 6).

Groups of six Swiss male mice (18–22 g) were injected in the right gastrocnemius muscle with total venom or PLA_2_ (50 mg/50 mL of PBS) or PBS alone (50 mL). After 3 h, blood was collected from the tail in heparinized capillary tubes and centrifuged for plasma separation. Activity of creatine kinase (CK) was then determined using 4 mL of plasma, which was incubated for 3 min at 37°C with 1.0 mL of the reagent according to the kinetic CK-UV protocol from Bioclin, Brazil. The activity was expressed in U/L, where one unit corresponds to the production of 1 mmol of NADH per minute (Figure 6). Myotoxic activity was expressed as mean ± SD of three independent experiments and each experiment was carried out in triplicate (*n* = 6) (Figure 5) [17, 18, 28, 29, 31, 33, 34].

### 2.5. Structural Analysis

#### 2.5.1. MALDI-TOF Analysis of CoaPLA_2_

The molecular mass of CoaPLA_2_ was analyzed by matrix-assisted laser desorption/ionization-time-of-flight (MALDI-TOF) mass spectrometry using a MALDI-TOF/TOF—Proteomics Analyzer 4800 (Applied Biosystems). Sample treatment: CoaPLA_2_ was analyzed at a concentration of 0.8 *μ*L of matrix and mixed with 0.5 *μ*L of sample on the MALDI plate. Samples were allowed to dry before analysis. The matrix consisted of 10 mg/mL sinapinic acid in 50% acetonitrile/miliQ water (v/v) and 0.1% trifluoroacetic acid (TFA). To calibrate the apparatus, a BSA standard solution was prepared following the same procedure, and 4 pmols were analyzed under the same conditions.

#### 2.5.2. Sequencing Procedure

*(1) In Solution Digestion*. Proteins were reduced by treatment with a solution of 20 mM DTT (Dithiothreitol) in 50 mM NH_4_HCO_3_ for 1 h at 30°C and alkylated with a solution of 150 mM iodine acetamide in 50 mM NH_4_HCO_3_ for 1 h at 30°C. The sample was then digested overnight at 37°C with trypsin (sequencing grade modified, Promega). Tryptic peptides were then cleaned-up with a Proxeon Stage tip. Peptides were eluted from the tip with 70% acetonitrile/0.1% trifluoroacetic acid. The eluted peptides were dried in a vacuum centrifuge and resuspended in 1% formic acid for LC-MS/MS analysis [45, 46]. 

*(2) LC-MS/MS Analysis*. Mass spectrometry was performed in a NanoAcquity (Waters) HPLC coupled to an OrbitrapVelos mass spectrometer (Thermo Scientific). An aliquot of the tryptic digest was injected and separated in a C18 reverse phase column (75 *μ*m Oi, 10 cm, nano-Acquity, 1.7 *μ*m BEH column, Waters). Bound peptides were eluted from the column with the following gradient: 1 to 40% B in 20 minutes, followed by a gradient of 40 to 60% B in 5 minutes; flow was 250 nL/min (A: 0.1% formic acid in water; B: 0.1% formic acid in acetonitrile). Eluted peptides were ionized in an emitter needle (PicoTipTM, New Objective). The spray voltage applied was 1900 V. Peptide masses (*m/z*: 300–1700) were measured in full scan in the Orbitrap at a resolution of 60,000 at *m/z* 400. The 5 most abundant peptides (minimum intensity of 1500 counts) were selected from each MS scan and fragmented in the HCD collision cell using a normalized collision energy (NCE) of 40% with nitrogen as the collision gas. Fragments were detected in the Orbitrap with a resolution of 7500 FWHM at 400 *m/z*. Raw data were collected with Thermo Xcalibur (v.2.1.0.1140). We obtained eight fragments, as shown in Table 1 [45, 46]. 

*(3) Database Search*. Raw data were analyzed using Proteome Discoverer (v.1.3.0.339) software. A search was run with the search engine MASCOT against NCBInr Serpentes database. The Percolator node was used in the Proteome Discoverer Mascot search in order to discriminate correct from incorrect peptide spectrum matches using the *q*-value (FDR) to improve the number of confidently identified peptides at a given false discovery rate. The results have been filtered so only high confidence peptides (FDR ≤ 0.01) have been considered for identification results [46, 47]. 

### 2.6. Tridimensional Structure Modeling

#### 2.6.1. Threading Modeling

The initial 3D model of PLA_2_ from *C. oreganus abyssus* venom was generated employing the threading modeling technique [39, 40, 48–57] implemented in the HHPred webserver (http://toolkit.tuebingen.mpg.de/hhpred). Initially, we generated 31 primary structure alignments using X-Ray structures available at the Protein Data Bank (PDB, http://www.rcsb.org/pdb/home/home.do) employing the global alignment option and scoring the predicted secondary structure alignment. From the 5 best ranked alignments, we evaluated the crystallographic quality (resolution and *R*-free value, Ramachandran distribution, and b-factor) of the target proteins. The phospholipase A_2_ from *Crotalus durissus terrificus* (PDB ID: 3R0L chain D) [58] was chosen as a template to thread the modeling, presenting 51.1% of identity and 100% of hit probability, with an *E*-value of 2.1^−41^ and score of 255.95.

#### 2.6.2. Molecular Dynamics

A molecular dynamics (MD) simulation was carried out aiming to refine the constructed PLA_2_ model. The MD simulation was performed employing GROMACS 4.5.4 package [48, 49], running with a 8G RAM Intel Xeon processor. Explicit water molecules were used employing the Simple Point Charge (SPC) model [50]. Protonation states of charged groups were set according to pH 7.0 and counter ions were added to neutralize the system. Gromos force field [51] was chosen to perform the MD simulation. The MD simulation was performed at constant temperature and pressure in a periodic truncated cubic box with a volume that was equal to 259.14 nm^3^ and at a minimum distance of 5 Å between any atom of the protein and the box wall. Sodium ions were added as counter ions to neutralize the system.

Initially, an energy minimization using the steepest descent algorithm was performed. After, 20 ps of MD simulation with position restraints applied to the protein were performed at 300 K to relax the system. Finally, an unrestrained MD simulation was performed at 300 K during 10 ns to assess the stability of the structures. During the simulation, temperature and pressure (1.0 bar) were maintained by coupling to an external heat and an isotropic pressure bath.

#### 2.6.3. Structural Analysis and Validation

After the MD simulation, several tools of structural analysis contained in the GROMACS package were employed to evaluate the final 3D model. All figures were generated employing PyMOL 0.99c software [52]. Other validation methods were also used, such as a pseudoenergy profile, which was analyzed with Verify 3D [53, 54] and ProSA-web [55, 56], as well as the Ramachandran plot [57], ERRAT program [39], and ANOLEA web server [40].

### 2.7. Statistical Analysis

Results are presented as mean ± SD obtained with the indicated number of animal samples or in vitro assays. The statistical significance of differences between groups was evaluated using the Student's unpaired *t*-test and ANOVA analysis of variance. Significance levels were considered at a confidence interval of 0.1 > *P* > 0.05.

## 3. Results and Discussion

### 3.1. Isolation and Purification of the Phospholipase A_2_ from *Crotalus oreganus abyssus* (CoaPLA_2_)

The process used to obtain the pure protein (CoaPLA_2_) is shown in Figure 2. Gel filtration (Figure 2(a)) demonstrated the presence of fraction II containing PLA_2_ activity, which was further purified.  Figure 2(b) shows the HPLC profile obtained using a reverse phase C18 column and the detachment of the peak containing CoaPLA_2_. This peak was also further purified by rechromatography and subjected to electrophoresis (SDS-PAGE and PAGE). As shown in Figure 2(c), the purification process was efficiently purified. Nondenaturation electrophoresis showed that CoaPLA_2_ was a dimeric protein with a molecular mass of approximately 28 kDa (lines 3 and 6), but under denaturing conditions, it was a monomer with a molecular mass of approximately 14 kDa (lines 2 and 5). This information was subsequently confirmed by MALDI-TOF mass spectrometry.

### 3.2. Biochemical Characterization of CoaPLA_2_

We biochemically analyzed and characterized CoaPLA_2_. Figure 3 shows phospholipase A_2_ activity under several conditions. We measured the phospholipase A_2_ activity of the isolated enzyme and total venom (using two different methods related to substrate type), as well as the optimal temperature and pH, and the influence of ions on the activity of the enzyme. Figures 3(a) and 3(b) show the PLA_2_ activity of the CoaPLA_2_ and of the total venom from *Crotalus oreganus abyssus*, *Crotalus viridis viridis,* and *Crotalus viridis nuntius*.  Figure 3(a) shows that the phospholipase A_2_ activity (using the nonmicellar substrate, 4-nitro-3-octanoyloxy benzoic acid) of the CoaPLA_2_ is approximately 48 nmol/min/mg, while the total venom from *Crotalus oreganus abyssus* has a PLA_2_ activity (approximately 22.5 nmol/min/mg) that is very different to the PLA_2_ activity of venom from *Crotalus viridis viridis* and *Crotalus viridis nuntius* (approximately 53 and 9 nmols/min/mg, resp.). Conversely, using the micellar substrate, 1-hexadecanoyl-2-(1-pyrenedecanoyl)-sn-glycero-3-phosphoglycerol, Figure 3(b) shows that the activity of the CoaPLA_2_ was approximately 590 *μ*mol/min/mg, while the total venom from *Crotalus oreganus abyssus* has a PLA_2_ activity (approximately 276 *μ*mol/min/mg) that was significantly different to the PLA_2_ activity of venom from *Crotalus viridis viridis* and *Crotalus viridis nuntius* (approximately 606 and 51 *μ*mols/min/mg, resp.). Both methods clearly demonstrate that the venom from *Crotalus oreganus abyssus* that we used to isolate CoaPLA_2_ was not derived from* Crotalus viridis viridis* or *Crotalus viridis nuntius*.

Interestingly, the enzymatic activity, obtained by Tsai et al., 2003, when cloning E6d was around 680 *μ*mol/min/mg and when using the micellar substrate L-dipalmitoyl-glycero-phosphatidyl-choline, being relatively close to the value found in the present study. We used a different, but similar, micellar substrate (1-hexadecanoyl-2-(1-pyrenedecanoyl)-sn-glycero-3-phosphoglycerol) and obtained a value of enzymatic activity of approximately 590 *μ*mol/min/mg.

The optimal temperature of CoaPLA_2_ was determined to be 37.3°C (Figure 3(d)) and optimal pH was 7.9 (Figure 3(c)). These values are in accordance with other PLA_2_ measurements described in the literature [12–34]. The influence ions on the enzyme activity was determined in the presence and absence of Ca^2+^ and other divalent cations (also in the presence and absence of Ca^2+^).  Figure 3(e) shows that the PLA_2_ activity of CoaPLA_2_ is calcium-dependent. In the presence of 10 mM calcium, the PLA_2_ activity was 45.8 nmols/min/mg. When the calcium concentration was 1 mM calcium, the phospholipase A_2_ activity was slightly reduced to 38.1 nmols/min/mg. A complete absence of calcium ions drastically reduced the enzyme activity to values of approximately 3 nmols/min/mg.

When 10 mM of other divalent cations (Mg^+2^, Cd^+2^ and Mn^+2^) was employed, the activity of the PLA_2_ was completely suppressed. However, phospholipase A_2_ activity was recovered when Ca^+2^ was mixed with these divalent cations (Mg^+2^, Cd^+2^ and Mn^+2^), both at concentrations of 1 mM and 10 mM (Figure 3(e)).

### 3.3. Biological Characterization of CoaPLA_2_

The biological characterization of CoaPLA_2_, isolated from *Crotalus oreganus abyssus,* was carried out using measurements of lethal activity (LA_50%_—dose that causes death in 50% of animal subjects), edema-inducing and myotoxic activities. We tested the lethal activity (LA_50%_) of CoaPLA_2_ and of the total venom of *Crotalus oreganus abyssus, Crotalus viridis viridis, and Crotalus viridis nuntius.* Figure 4 shows that LA_50%_ of the venom from *Crotalus oreganus abyssus* is approximately 2.2 ± 0.4 *μ*g of venom/g of mouse and this value is bigger in relation to *C. v. viridis* and *C. v. nuntius*. CoaPLA_2_ has a LA_50%_ at a dose of about 1.8 *μ*g ± 0.2 of venom/g mouse weight and was higher than that of the total venom of *C. v. viridis* and *C. v. nuntius*. The total *C. o. abyssus* venom is more lethal than CoaPLA_2_ alone, as venom contains other enzymes that also exhibit lethality, such as serine proteases and metalloproteases [12–34].

The edema-inducing activity of CoaPLA_2_ was measured using different dosages of the enzyme (25, 50, and 100 *μ*g). From Figure 5, we can see that the edema-inducing activity of CoaPLA_2_ is dose-dependent. The increase in the amount of enzyme increases the percentage of edema formed, principally in the first 24 h. After this time, this edema-inducing activity is significantly reduced and the edema is suppressed.

Similarly to the edema-inducing activity results obtained, the myotoxic activity induced by CoaPLA_2_ was also dose-dependent. When we increased the quantity of CoaPLA_2_ (25, 50 and 100 *μ*g) its myotoxic effects were augmented.

The phospholipases A_2_ are a group of enzymes present in most venoms or oral secretions of snakes. In addition to the digestive function of the prey, these enzymes interfere with the physiological processes and cause many pharmacological and pathophysiological effects, such as neurotoxic, cardiotoxic, anticoagulant, antiplatelet, hemolytic, hemorrhagic, and inflammatory activities [59–61].

Both the crude venom of *Crotalus oreganus abyssus* and the isolated CoaPLA_2_, were able to induce experimental toxicity, such as myonecrosis, edema, and mortality. Due to the neurotoxic potential of this kind of snake, it was observed that the LD_50%_ of the crude venom of *C. oreganus abyssus* and its CoaPLA_2_ showed low values of lethal doses, when compared to *Bothrops* genera venoms and its isolated PLA_2_s [36].

The CoaPLA_2_ also induced myotoxic activity, similarly to other PLA_2_s isolated from snake venoms. The myotoxicity was evaluated by the activity levels of creatine kinase (CK) in the plasma of animals. Creatine kinase is an enzyme used in muscular energy metabolism and, in cases of cell damage, is released and can be detected in plasma as a marker [36, 60]. The catalytic activity of the PLA_2_ on the membrane suggests an important role these enzymes in the toxicity of snake venoms (svPLA_2_s). The breakdown of phospholipids causes severe changes in the structural and functional integrity of the plasmatic membrane with a consequent influx of calcium ions [62], release of calcium-dependent proteases [63], activation of endogenous PLA_2_s [64], and mitochondrial collapse [65]. The sum of all these molecular changes could lead to cell death.

The CoaPLA_2_ was able to induce edema in mice paws. Local inflammation is a feature of poisoning by snakes of the subfamilies of Viperidae and Crotalidae [60, 61]. The catalytically active mechanism by which PLA_2_ induces edema is probably due to the release of precursors of eicosanoids due to the hydrolysis of phospholipids. Release of biogenic amines from mast cells is also proposed as a possible mechanism of induction of edema by PLA_2_ [66, 67].

### 3.4. Structural Characterization of CoaPLA_2_

**Figure 7:**
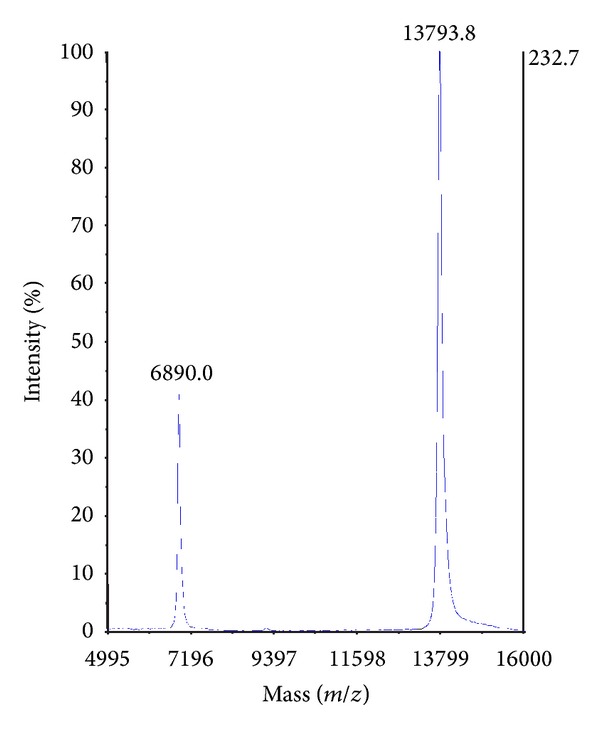
MALDI-TOF analysis of CoaPLA_2_. The molecular mass of CoaPLA_2_ (Da) was analyzed by matrix-assisted laser desorption/ionization-time-of-flight (MALDI-TOF mass spectrometry—Proteomics Analyzer 4800). The peak with a molecular mass of 13793.8 Da corresponds to s monoisotopic ion (*m/z*, where *z* = +1) and the peak with a molecular mass of 6890.0 Da corresponds to a diisotopic ion (*m/z*, where *z* = +2).

The molecular mass of CoaPLA_2_ was analyzed by MALDI-TOF mass spectrometry (Figure 7) and the mass determined was 13.793.8 Da. However, interestingly, Tsai et al. [37] found the cDNA E6d product to present a molecular mass of 13.782 Da. In addition, Tsai et al. [37], based on a phylogenetic analysis, also found that all cDNA products from all specimens studied showed biological relationships among themselves, with the exception of the cDNA E6d product. Authors inferred and suggested that the specimen, initially considered as *Crotalus viridis viridis*, which produced the E6d cDNA, in reality belonged to a distinct population present in Southwestern Arizona.

To extend our structural study of CoaPLA_2_, we determined its primary structure using LC/MS-MS, after in-solution digestion (tryptic digestion). The fragments obtained were analyzed using the Proteome Discoverer (v.1.3.0.339) software, where a search was run with the MASCOT engine against the NCBInr Serpentes database. The search found eight fragments (Table 1). The analysis using the ClustalW multiple sequence alignment showed that fragments recovered from the PLA_2_ sequence produced by the cDNA E6d and described by Tsai et al. [37] displayed 94% sequential homology (Table 2), except for a unique fragment that was not found (VTDCNPK). From Table 2 we can see that this fragment is extremely conserved in all sequences analyzed and in the model proposed by us (Figure 10). We have inserted the sequence VTDCNPK in the gap of the fragment as not found, as shown in the E6D sequence.

The comparison between the E6D sequence and the CoaPLA_2_ sequence obtained is interesting because both are exactly equal, except for the VTDCNPK fragment (not found in this study). Tsai et al., [37] suggested that the specimen, initially considered as *Crotalus viridis viridis*, may be a distinct population present in Southwestern Arizona, considered the natural habitat of *Crotalus oreganus abyssus* (Figure 1(b)). Thus, we infer that, probably, the specimen used by Tsai et al., [37] that produced the E6D cDNA was actually a *Crotalus oreganus abyssus* snake, and not a *Crotalus viridis viridis*. In addition to this information, and to enforce our conclusion, it should be remembered that the value of the enzymatic activity of CoaPLA_2_ found in this work is very near to the value found by Tsai et al., 2003 (around 680 *μ*mol/min/mg for the E6d clone and approximately 590 *μ*mol/min/mg for CoaPLA_2_).

This fact supports the need for more studies on *Crotalus oreganus *(all subspecies) because for many years all subspecies of *Crotulus viridis* and *Crotalus oreganus* were treated as a single serpent specimen. However, as subsequent studies have shown, the old classification was incorrect and the “old” *Crotalus viridis* can in fact be divided into two subspecies of *Crotalus viridis* (*viridis* and *nuntius*) and seven subspecies of *Crotalus oreganus* (*abyssus, lutosus, concolor, oreganus, helleri, Cerberus, and caliginis*).

### 3.5. Molecular Modeling

**Figure 8:**
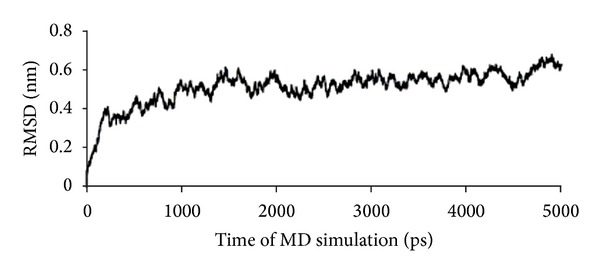
RMSD values of the PLA_2_ backbone atoms along with the MD simulation.

To increase the understanding of the CoaPLA_2_ structure, we conducted molecular modeling studies using Molecular Dynamics (MD) simulation. We calculated the values of root mean squared distance (RMSD) considering the protein backbone atoms, which are displayed in Figure 8. When analyzing these results, we noted that the PLA_2_ model was stabilized after approximately 1300 ps of simulation.

**Figure 9:**
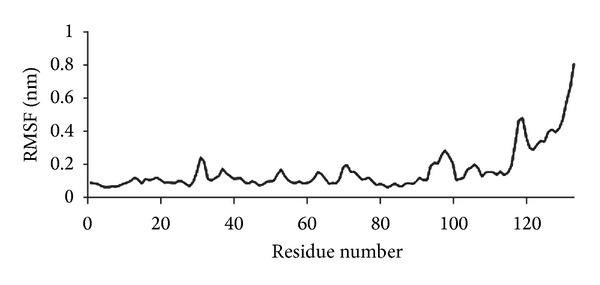
RMSF values per residue after protein stabilization.

**Figure 10:**
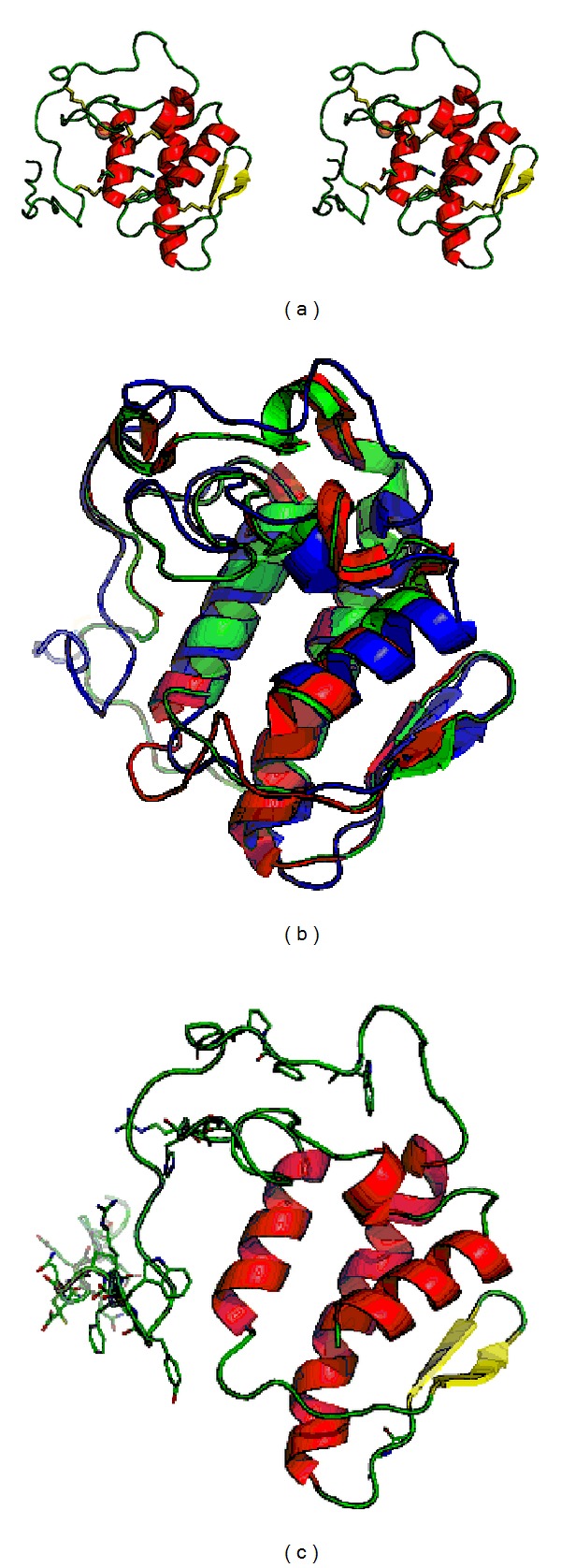
(a) Stereoview of the final model; (b) superimposition of the initial (green), final (blue), and template (red) models; (c) residues with high energy predicted by ANOLEA are displayed in wireframe model.

From the root mean squared fluctuation (RMSF) values of the alpha carbons per residue, we can see that the fluctuation of PLA_2_ residues from 1300 ps to 5000 ps is very low (except for the residues in the terminal loop, 119–133), indicating that there are no significant changes in the conformation of the residues (Figure 9).

Comparing the initial 3D model and the final model obtained after the MD simulation, it can be easily seen that the MD simulation is fundamental to refine the PLA_2_ model. The ProSA energy profile indicates that both initial and final models have energy values per residue of lower than 0, indicating a good pseudoenergy profile.  Table 3 displays the results obtained from ANOLEA, ERRAT, Verify 3D, ProSA, and Ramachandran analyses for the initial and final models.

The ANOLEA results indicate that the MD simulation decreased the number of high energy residues by 8%. With all high energy residues of the final model being located in the loop region (Figure 9(c)). The ERRAT results indicate that the MD simulation improved the quality of the structural model from 64% to 99%. From the Verify 3D results, the initial model had 17 residues with poor structural quality (score lower than 0) and all residues of the final model had score values of higher than 0. Finally, the Ramachandran plot analysis indicates that both initial and final models had 3 and 2 glycine residues located at an outlier region, respectively. Figure 9(a) shows the Stereoview of the final model. Figure 9(a) shows that our model displays the typical phospholipase conformation, containing three parallel *α*-helixes and a *β*-wing (one double-stranded antiparallel *β*-sheet) [20].

Data reinforce the necessity of rearranging and clarifying all information available regarding the two subspecies of *Crotalus oreganus* and *Crotalus viridis*, considering the length of time during which these species were considered as one and the same.

## Figures and Tables

**Table 1:** Peptide fragments obtained by sequencing procedure. The CoaPLA_2_ was reduced and digested overnight with trypsin. Eluted tryptic peptides were dried in a vacuum centrifuge and resuspended in 1% formic acid for LC-MS/MS analysis.

Number	Peptide fragment	Molecular mass (MH^+^-Da)
1	SLVQFEMLIMKVAKR	1793.01230
2	SGLFSYSAYGCYCGWGGHGR	2241.91338
3	PQDATDHCCFVHDCCYGK	2269.83194
4	TASYTYSEENGEIVCGGDDPCKK	2580.07798
5	QVCECDR	966.37218
6	VAAICFR	836.44066
7	DNIPTYDNK	1079.49590
8	FPPENCQEEPEPC	1632.62198

**Table 2:** Multiple alignment of fragment of CoaPLA_2_.

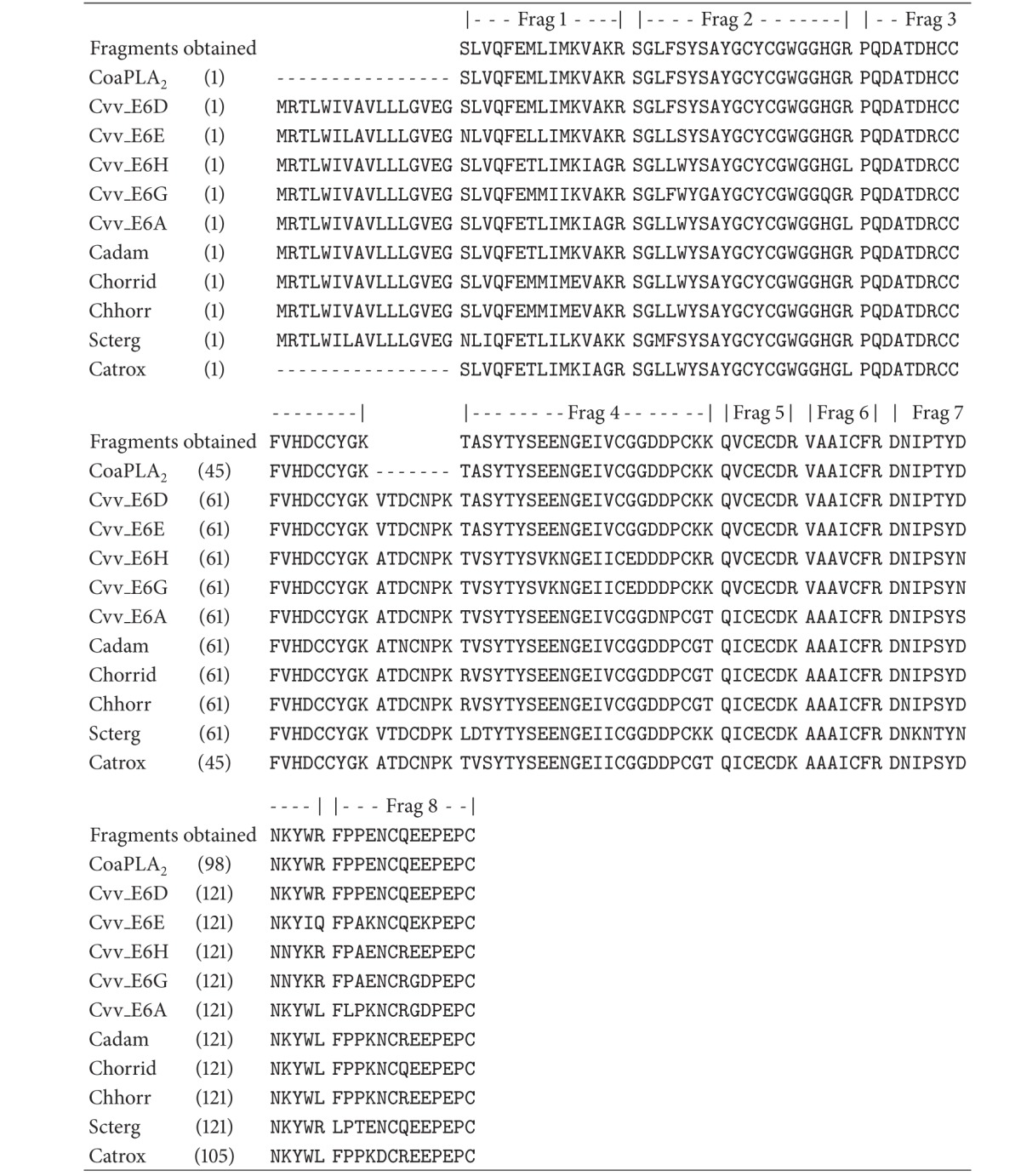

CoaPLA_2_: Crotalus oreganuis abyssus; Cvv_E6D, Cvv_E6E, Cvv_E6H, Cvv_E6G, and Cvv_E6A (cDNA from Crotalus viridis viridis [37]), Cadam: Crotalus adamanteus [39]; Chorrid and Chhorrid: Croatus horridus and Crotalus horridus horridus [40]; Scterg: Sistrurus catenatus tergeminus [41]; Catrox: Crotalus atrox [42].

**Table 3:** Results for ANOLEA, ERRAT, Verify 3D, ProSA, and Ramachandran analyses for the initial and final models.